# MnTE-2-PyP Attenuates TGF-*β*-Induced Epithelial-Mesenchymal Transition of Colorectal Cancer Cells by Inhibiting the Smad2/3 Signaling Pathway

**DOI:** 10.1155/2019/8639791

**Published:** 2019-02-25

**Authors:** Yu Yang, Pei Zhang, Ruicheng Yan, Qi Wang, Erhu Fang, Hongxue Wu, Shijun Li, Haiyan Tan, Xing Zhou, Xianxiong Ma, Yu Tang, Yongming Huang, Rui Deng, Ying Liu, Shilun Tong, Zhihua Wang, Rebecca E. Oberley-Deegan, Qiang Tong

**Affiliations:** ^1^Department of Gastrointestinal Surgery I Section, Renmin Hospital of Wuhan University, Wuhan 430060, China; ^2^Department of Gastrointestinal Surgery, Union Hospital, Tongji Medical College, Huazhong University of Science and Technology, Wuhan 430022, China; ^3^Department of Hepatobiliary Surgery, Union Hospital, Tongji Medical College, Huazhong University of Science and Technology, Wuhan 430022, China; ^4^Central Laboratory, Renmin Hospital of Wuhan University, Wuhan 430060, China; ^5^Department of Biochemistry and Molecular Biology, University of Nebraska Medical Center, Omaha, NE 68198, USA

## Abstract

**Background:**

As a key step in enhancing cancer cell invasion and metastasis, epithelial-mesenchymal transition (EMT) plays an important role in colorectal cancer progression. EMT is triggered by a variety of signaling pathways, among which the transforming growth factor *β* (TGF-*β*) signaling pathway has been implicated as a primary inducer. Accumulating evidence demonstrates that MnTE-2-PyP (chemical name: manganese(III) *meso*-tetrakis-(N-ethylpyridinium-2-yl), a superoxide dismutase (SOD) mimetic, inhibits TGF-*β* signaling; however, its ability to inhibit TGF-*β*-induced EMT in colorectal cancer has not yet been explored.

**Methods:**

To verify our hypothesis that MnTE-2-PyP attenuates TGF-*β*-induced EMT, human colorectal cancer cells were treated with TGF-*β* in the presence or absence of MnTE-2-PyP. Cells were analyzed by several techniques including western blotting, real-time quantitative PCR, transwell assay, and wound healing assay.

**Results:**

MnTE-2-PyP reverses cell phenotypes induced by TGF-*β* in colon cancer cells. MnTE-2-PyP treatment significantly reduced the expression of mesenchymal markers but maintained epithelial marker expression. Mechanistically, MnTE-2-PyP suppressed the phosphorylated Smad2/3 protein levels induced by TGF-*β* in SW480 cells, but MnTE-2-PyP failed to suppress TGF-*β*-induced Slug and Snail expression in colorectal cells. Furthermore, MnTE-2-PyP effectively suppressed TGF-*β*-mediated cell migration and invasion and the expression of matrix metalloproteinase 2 (MMP-2) and matrix metalloproteinase 9 (MMP-9) in colorectal cells.

**Conclusion:**

Taken together, we provide an in-depth mechanism by which MnTE-2-PyP inhibits colorectal cancer progression, supporting an important role for MnTE-2-PyP as an effective and innovative antitumor agent to enhance treatment outcomes in colorectal cancer.

## 1. Introduction

Colorectal cancer is one of the deadliest cancers worldwide because of its local invasion and distant metastasis [1]. As a key step in enhancing cancer cell invasion and metastasis, EMT refers to the reprogramming of epithelial cells to a mesenchymal-like phenotype [2–4]. EMT is mediated by a set of transcription factors such as Slug, Snail, Twist, and Zeb1/2, which can inhibit the expression of the epithelial marker E-cadherin and induce the expression of mesenchymal markers including N-cadherin, vimentin, and fibronectin [1, 5–7]. A variety of signaling pathways can trigger EMT by regulating those transcription factors, specifically the TGF-*β* signaling pathway that has been implicated as a primary inducer [8–11]. In TGF-*β* signaling pathways, the activated Smad2 and Smad3 combine with Smad4 to form Smad transcription factor complexes, which subsequently shuttle into the nucleus to regulate gene expression. Also, TGF-*β* induces EMT through non-Smad pathways such as MAPK, PI3K/AKT, GTPases, NF-*κ*B, and HIF-1 pathways. TGF-*β* signaling pathway components, including the ligands, receptors, and Smad proteins, are regulated by a variety of mechanisms [11–15]. Oxidative stress has been proposed as one of these mechanisms that enhance TGF-*β*-induced EMT in numerous types of cells [16, 17].

Superoxide dismutase mimics (SODm) are a group of synthetic compounds that are able to mimic the functions of native superoxide dismutases. Meanwhile, manganese porphyrins (MnPs) have emerged as one of the most promising classes of SODm [18]. It has been established that the manganese(III) porphyrin MnTnHex-2-PyP(5+) reduces the migration of doxorubicin-treated breast cancer cells [19]. Besides, it was observed in a glioma mouse study [20] that MnP+radiation vs. radiation treatment alone downregulated metastatic pathways. Mn(III) *meso*-tetrakis (N-ethylpyridinium-2-yl) porphyrin (MnTE-2-PyP(5+), AEOL10113, and BMX-010) is another Mn porphyrin analog which scavenges reactive oxygen species (ROS), including superoxides, lipid peroxides, and peroxynitrite [5, 21, 22]. Accumulating evidence demonstrates that MnTE-2-PyP plays a critical role in TGF-*β* signaling regulation [5, 23–25]. Treatment with MnTE-2-PyP decreases the TGF-*β* levels in a radiation-induced lung injury model [24, 25], which is similar to the result of another SOD mimic MnTDE-2-ImP(5+) [26]. The effect of MnTE-2-PyP on TGF-*β*, IL4, and IL13 secreted by activated splenocytes was also observed in a mouse model of prostate cancer [27]. Jackson et al. have shown that the macrophage respiratory burst, more specifically superoxide anion, regulates macrophage production of TGF-*β*1, which can be suppressed by the SOD-like action of MnTE-2-PyP [23]. We have previously reported that MnTE-2-PyP significantly inhibits the TGF-*β*1 signaling pathway by downregulating the expression of TGF*β*RII, which protects against radiation-induced mouse primary prostate fibroblast damage. Due to the downregulation of the expression of TGF*β*RII, the extracellular TGF-*β* could not bind to its receptor. As a consequence, the phosphorylation and total expression of Smad2 are reduced by MnTE-2-PyP [5]. Additionally, MnTE-2-PyP treatment downregulates the total protein levels of Smad3 and Smad4 [5]. Since activated TGF-*β* signaling and enhanced ROS contribute to EMT and excessive extracellular matrix (ECM) deposition leading to fibrosis, it is possible that MnTE-2-PyP protects against radiation-induced mouse primary prostate fibroblast damage by inhibiting TGF-*β*-induced EMT [5]. However, the ability of MnTE-2-PyP to inhibit TGF-*β*-induced EMT in colorectal cancer has not been examined.

In this study, we report that MnTE-2-PyP reverses TGF-*β*-mediated EMT in colorectal cancer cells *in vitro*. MnTE-2-PyP inhibited TGF-*β*-induced expression of EMT markers and morphological changes in colorectal cancer cells. Mechanistically, MnTE-2-PyP attenuated TGF-*β*-induced EMT in colorectal cancer cells by inhibiting the Smad2/3 signaling pathway. However, MnTE-2-PyP did not suppress TGF-*β*-induced Slug and Snail expression in colorectal cells. MnTE-2-PyP effectively suppressed TGF-*β*-induced cell migration and invasion and the expression of matrix metalloproteinase 2 (MMP-2) and matrix metalloproteinase 9 (MMP-9) in colorectal cancer cells. These findings are crucial in determining how MnTE-2-PyP can regulate cancer cell invasion and metastasis and how it can be better used for the treatment of colorectal cancer.

## 2. Methods

### 2.1. Cell Culture and TGF-*β* Treatment

The LOVO, HT29, and SW480 human colorectal cancer cell lines were purchased from the American Type Culture Collection (ATCC, Manassas, VA, USA) and cultured in RPMI 1640 (Gibco-Life Technologies, Grand Island, NY, USA) supplemented with 10% fetal bovine serum (FBS) at 37°C with 5% CO_2_. In order to induce the EMT process, human colorectal cancer cells were treated with 5 ng/ml TGF-*β* (R&D Systems, Minneapolis, MN, USA) for 48 h. MnTE-2-PyP was a gift from Dr. Rebecca E. Oberley-Deegan at the University of Nebraska Medical Center, Omaha, NE, USA.

### 2.2. Real-Time Quantitative PCR

The total RNA was extracted from the cells using the TRIzol Reagent (Invitrogen, Carlsbad, CA, USA) according to the manufacturer's instructions. The residual traces of DNA were removed from the RNA samples with DNase I. RNA was reverse transcribed to cDNA with 2 *μ*g of total RNA, using Moloney murine leukemia virus reverse transcriptase and random primers. The cDNA was then amplified by PCR using specific primers. The sequences for the amplification of human MMP-2 were 5′-TACAGGATCATTGGCTACACACC-3′ (forward) and 5′-GGTCACATCGCTCCAGACT-3′ (reverse). The sequences for the amplification of human MMP-9 were 5′-TGTACCGCTATGGTTACACTCG-3′ (forward) and 5′-GGCAGGGACAGTTGCTTCT-3′ (reverse). The sequences for the amplification of human Slug were 5′-TGTGACAAGGAATATGTGAGCC-3′ (forward) and 5′-TGAGCCCTCAGATTTGACCTG-3′ (reverse). The sequences for the amplification of human Snail were 5′-TCGGAAGCCTAACTACAGCGA-3′ (forward) and 5′-AGATGAGCATTGGCAGCGAG-3′ (reverse). The sequences for the amplification of human Twist were 5′-GTCCGCAGTCTTACGAGGAG-3′ (forward) and 5′-GCTTGAGGGTCTGAATCTTGCT-3′ (reverse). The sequences for the amplification of human Zeb1 were 5′-CAGCTTGATACCTGTGAATGGG-3′ (forward) and 5′-TATCTGTGGTCGTGTGGGACT-3′ (reverse). The sequences for the amplification of human Zeb2 were 5′-CAAGAGGCGCAAACAAGCC-3′ (forward) and 5′-GGTTGGCAATACCGTCATCC-3′ (reverse). 18S rRNA was used as an external endogenous standard. The sequences for the amplification of 18S rRNA were 5′-CGGCTACATCCAAGGAA-3′ (forward) and 5′-GCTGGAATTACCGCGGCT-3′ (reverse).

### 2.3. Western Blotting

Total protein was isolated from cells, and protein concentration was determined by a Coomassie protein assay (Pierce, Rockford, IL, USA). The protein for each cell lysate was separated using SDS-PAGE and electrotransferred to polyvinylidene fluoride (PVDF) membranes. After blocking in skim milk at room temperature for 30 min, the membranes were incubated with specific primary antibodies at 4°C overnight. Next, the membranes were incubated with a secondary antibody (1 : 5000 dilution, Wuhan Boster Biological Technology Ltd., Wuhan, China) at room temperature for 2 h. *β*-Actin was used as the internal control. Finally, the signals were detected using ECL reagents (Pierce, Rockford, IL, USA). The following antibodies were used in western blot experiments: TGF-*β* (1 : 1000, BD Biosciences, San Diego, CA, USA), E-cadherin (1 : 1000, BD Biosciences, San Diego, CA, USA), N-cadherin (1 : 500, Santa Cruz Biotechnology Inc., Santa Cruz, CA, USA), occludin (1 : 1000, Abcam, Cambridge, MA, USA), vimentin (1 : 1000, Abcam, Cambridge, MA, USA), Smad2 (1 : 1000, Cell Signaling Technology, Danvers, MA, USA), P-smad2 (1 : 1000, Cell Signaling Technology, Danvers, MA, USA), Smad3 (1 : 1000, Cell Signaling Technology, Danvers, MA, USA), P-smad3 (1 : 1000, Cell Signaling Technology, Danvers, MA, USA), Slug (1 : 1000, Cell Signaling Technology, Danvers, MA, USA), Snail (1 : 1000, Cell Signaling Technology, Danvers, MA, USA), Twist (1 : 1000, Cell Signaling Technology, Danvers, MA, USA), Zeb1 (1 : 1000, Abcam, Cambridge, MA, USA), Zeb2 (1 : 1000, Abcam, Cambridge, MA, USA), GAPDH (1 : 1000, Cell Signaling Technology, Danvers, MA, USA), MMP-2 (1 : 1000, Abcam, Cambridge, MA, USA), and MMP-9 (1 : 1000, Abcam, Cambridge, MA, USA).

### 2.4. Transwell Assay

The invasive ability was evaluated using a transwell assay (8 *μ*m pore size; Corning Inc., Corning, NY, USA). The transwell top chambers were coated with 100 *μ*l of 1 mg/ml Matrigel (BD Biosciences, Franklin Lakes, NJ, USA). A cell suspension was prepared in serum-free medium; then, 1 × 10^4^ cells/well were seeded into the upper chamber of each insert and medium containing 10% FBS was placed into the lower chambers. After incubation for 24 h at 37°C in a 5% CO_2_ humidified incubator, noninvasive cells in the upper chamber were carefully removed with a cotton swab and invasive cells on the bottom were fixed and stained with 0.05% crystal violet. Cell numbers from five random fields were counted and expressed as the average number of cells/field. Three independent experiments were performed.

### 2.5. Wound Healing Assay

For the wound healing assay, all cell fractions were cultured in six-well plates. Cell monolayers were wounded by scratching with sterile micropipette tips. The migration of cells to cover the wound space was examined and photographed at indicated time points (0, 24, and 48 hours) in five random microscopic regions with the Carl Zeiss Axio Observer Z1 microscope. The migrated distance was analyzed by measuring distances between wounded margins at three randomly chosen points.

### 2.6. Statistical Analysis

Values were presented as means ± SD of measurements of at least three independently performed experiments to avoid possible variation of cell cultures. An ANOVA was performed, and a secondary test between two groups was determined by Student's *t*-test using the SPSS 19 software program. Statistical significance was defined as *P* value < 0.05.

## 3. Results

### 3.1. MnTE-2-PyP Inhibits TGF-*β*-Induced Morphological Changes in Colorectal Cancer Cells

**Figure 1:**
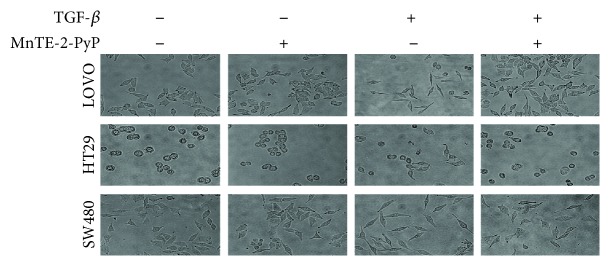
MnTE-2-PyP inhibits TGF-*β*-induced morphological changes in colorectal cancer cells. (a) The morphological changes of LOVO, HT29, and SW480 cells after exposure to MnTE-2-PyP alone, TGF-*β* alone, or MnTE-2-PyP combined with TGF-*β* were evaluated using light microscopy (×400 magnification). The cells were pretreated with MnTE-2-PyP (30 *μ*M) for 12 h and then treated with TGF-*β* (5 ng/ml) for two days. The cells lost their polarized epithelial phenotype with increased cell-cell connections and acquired mesenchymal traits after two days of TGF-*β* treatment. The cells became dispersed and assumed a fibroblast-like appearance. MnTE-2-PyP treatment inhibited TGF-*β*-induced morphologic changes significantly in LOVO, HT29, and SW480 cells. After treatment with MnTE-2-PyP, these cells kept a more epithelial-like appearance induced by TGF-*β*.

TGF-*β* has been implicated as a primary inducer of EMT in colorectal cancer. As shown in Figure 1, morphologic changes in LOVO, HT29, and SW480 cells were observed after two days of TGF-*β* (5 ng/ml) treatment. The cells lost their polarized epithelial phenotype with increased cell-cell close connections and acquired mesenchymal traits. They became dispersed and displayed a fibroblast-like appearance with a long shape and a central nucleus. To examine the impact of MnTE-2-PyP on TGF-*β*-induced morphological changes in colorectal cancer cells, cells were pretreated with MnTE-2-PyP for 12 h prior to stimulation with TGF-*β*. MnTE-2-PyP (30 *μ*M) treatment significantly inhibited TGF-*β*-induced morphologic changes in LOVO, HT29, and SW480 cells. After treatment with MnTE-2-PyP, these cells kept a more epithelial-like appearance even if induced by TGF-*β* (Figure 1). Based on these phenomena, we speculated that MnTE-2-PyP might attenuate TGF-*β*-induced EMT, which has been demonstrated to have an important role in cancer cell migration and invasion.

### 3.2. MnTE-2-PyP Attenuates TGF-*β*-Induced Expression of EMT Markers in Colorectal Cancer Cells

**Figure 2:**
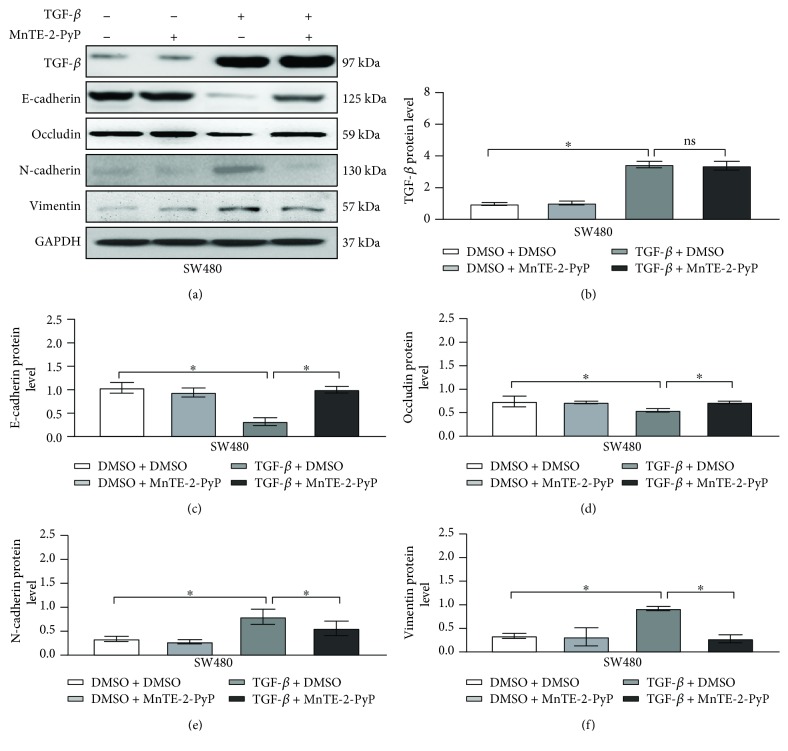
MnTE-2-PyP attenuates TGF-*β*-induced expression of EMT markers in colorectal cancer cells. (a) The SW480 cells were pretreated with MnTE-2-PyP (30 *μ*M) for 12 h and then treated with TGF-*β* (5 ng/ml) for 24 h. Western blotting analysis showed that TGF-*β* decreased the expression levels of the epithelial cell markers E-cadherin and occludin and increased the mesenchymal markers N-cadherin and vimentin in SW480 cells. MnTE-2-PyP suppressed the changes of markers related to EMT caused by TGF-*β* in SW480 cells. (b–f) Quantification of protein expression shown in (a) is normalized to GADPH. ^∗^*P* < 0.05 compared to the control group.

To determine whether MnTE-2-PyP regulates TGF-*β*-induced EMT in colorectal cancer cells, we evaluated the expression of EMT markers using western blot analysis. As expected, TGF-*β* decreased the expression levels of the epithelial cell markers, E-cadherin and occludin, and increased the mesenchymal markers, N-cadherin and vimentin, in SW480 cells (Figure 2). MnTE-2-PyP significantly suppressed the mesenchymal markers related to EMT caused by TGF-*β* while maintaining the epithelial cell markers in SW480 cells, suggesting a functional role of MnTE-2-PyP in inhibiting TGF-*β*-induced EMT in colorectal cancer cells.

### 3.3. MnTE-2-PyP Inhibits TGF-*β*-Induced EMT in Colorectal Cancer Cells Associated with Suppression of the Smad2/3 Signaling Pathway

**Figure 3:**
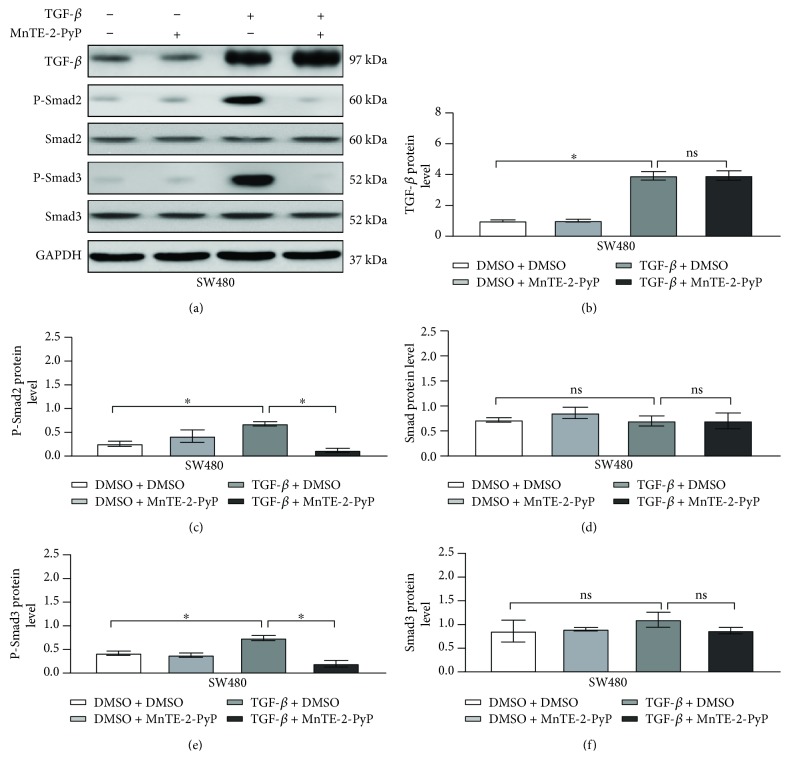
MnTE-2-PyP inhibits TGF-*β*-induced EMT in colorectal cancer cells associated with the suppression of the Smad2/3 signaling pathway. (a) The SW480 cells were pretreated with MnTE-2-PyP (30 *μ*M) for 12 h and then treated with TGF-*β* (5 ng/ml) for 24 h. Western blotting analysis showed that TGF-*β* induced phosphorylated Smad2/3 protein levels in SW480 cells, but the total Smad2/3 protein levels were not changed by TGF-*β* treatment in SW480 cells. MnTE-2-PyP suppressed the phosphorylated Smad2/3 protein levels induced by TGF-*β*. (b–f) Quantification of protein expression shown in (a) is normalized to GADPH. ^∗^*P* < 0.05 compared to the control group.

Next, we sought to determine the signaling mechanisms involved in the MnTE-2-PyP-mediated EMT process in colorectal cancer cells. TGF-*β*-induced activation of the receptor complex leads to the activation of Smad2 and Smad3. Phosphorylated Smad2 and Smad3 then form complexes with Smad4 and shuttle into the nucleus, where they bind to DNA and transcribe target genes [4, 13]. The addition of TGF-*β* significantly increased the phosphorylation of Smad2/3 in SW480 cells (Figures 3(a), 3(c), and 3(e)), but the total Smad2/3 protein levels were not altered by TGF-*β* treatment (Figures 3(a), 3(d), and 3(f)). MnTE-2-PyP markedly suppressed Smad2/3 protein phosphorylation induced by TGF-*β* (Figures 3(a), 3(c), and 3(e)). Consequently, these findings demonstrate a functional role by which MnTE-2-PyP inhibits the TGF-*β*-induced Smad2/3 signaling pathway in colorectal cancer cells.

### 3.4. MnTE-2-PyP Does Not Inhibit Slug and Snail Expression Induced by TGF-*β*

**Figure 4:**
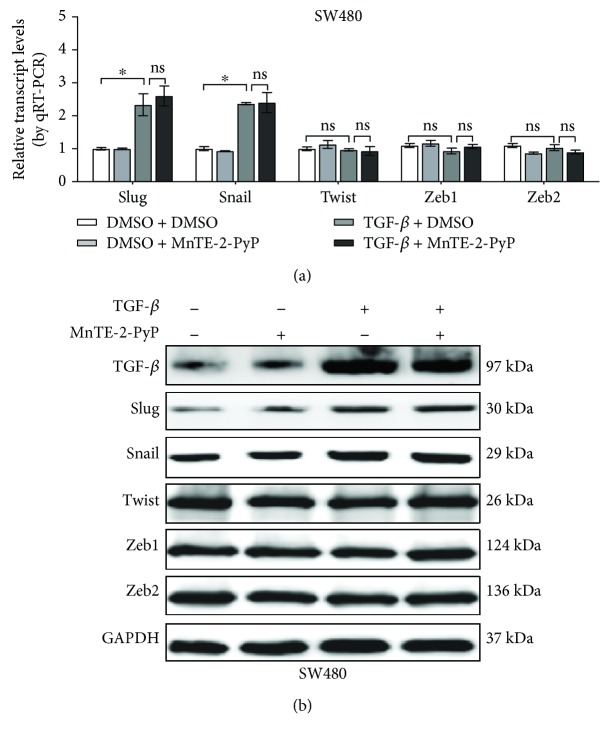
MnTE-2-PyP does not inhibit Slug and Snail expression induced by TGF-*β*. (a) The SW480 cells were pretreated with MnTE-2-PyP (30 *μ*M) for 12 h and then treated with TGF-*β* (5 ng/ml) for 24 h. PCR analysis of EMT-related transcription factors showed that TGF-*β* increased the expression levels of Slug and Snail, whereas no changes in the expression of Twist and Zeb1/2 were observed compared with those of the untreated controls in SW480 cells. Furthermore, the induction of Snail and Slug upon TGF-*β* treatment was not impaired in cells treated with MnTE-2-PyP. 18S rRNA was used as the reference gene. (b) Western blotting analysis showed that MnTE-2-PyP does not inhibit Slug and Snail expression induced by TGF-*β*. ^∗^*P* < 0.05 compared to the control group.

Because the downregulation of E-cadherin is mediated by transcription factors like Slug, Snail, Twist, and Zeb1/2, we investigated the regulation of these factors by the TGF-*β* signaling pathway. In TGF-*β*-treated SW480 cells, no changes in the expression of Twist and Zeb1/2 were observed compared with untreated controls (Figure 4). By contrast, TGF-*β* increased the expression levels of Slug and Snail (Figure 4). This is consistent with the recent publications that Snail and Slug are highly expressed in colorectal cancer [8, 10]. In addition, the contribution of Snail and Slug to the repression of E-cadherin and to EMT *in vivo* and *in vitro* has been demonstrated in colorectal cancer models [8, 10, 11, 28]. While the administration of MnTE-2-PyP could not reverse the changes in Snail and Slug expression induced by TGF-*β*, our results suggest that the induction of Snail and Slug upon TGF-*β* treatment is not impaired by MnTE-2-PyP.

### 3.5. MnTE-2-PyP Inhibits Colorectal Cancer Cell Migration and Invasion

**Figure 5:**
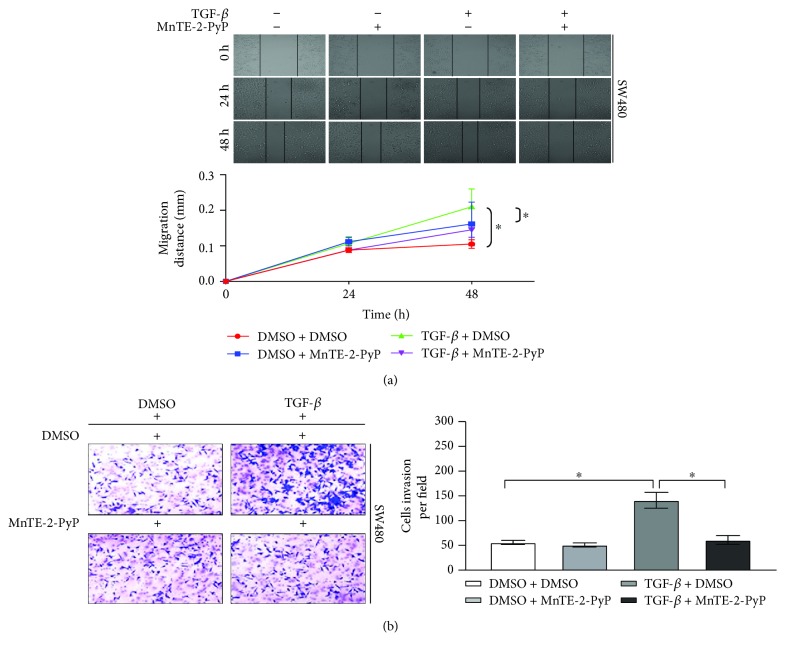
MnTE-2-PyP inhibits colorectal cancer cell migration and invasion. (a) The SW480 cells were pretreated with MnTE-2-PyP (30 *μ*M) for 12 h and then treated with TGF-*β* (5 ng/ml) for 24 h. Wound healing assays showed that MnTE-2-PyP inhibited TGF-*β*-induced cell migration. (b) The transwell assays showed that TGF-*β*-induced migration and invasion were also reduced with MnTE-2-PyP treatment.

TGF-*β* promotes migration and invasion, so we next investigated whether MnTE-2-PyP affects TGF-*β*-induced cell migration and invasion in colorectal cells. Wound healing and transwell assays were performed to evaluate the alteration of tumor cell migratory and invasive properties. MnTE-2-PyP substantially inhibited TGF-*β*-induced cell migration (Figure 5(a)), and TGF-*β*-induced transwell migration and invasion were also significantly reduced by MnTE-2-PyP (Figure 5(b)). These results indicate that MnTE-2-PyP suppresses TGF-*β*-induced colorectal cancer cell migration and invasion in vitro.

### 3.6. MnTE-2-PyP Reverses the Expression of MMP-2 and MMP-9 Induced by TGF-*β*

**Figure 6:**
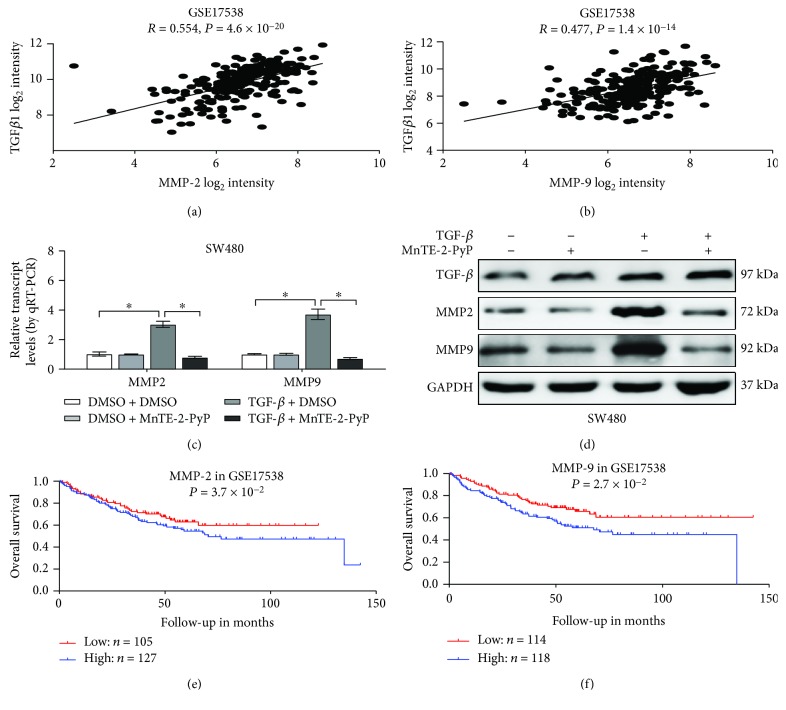
MnTE-2-PyP reverses the expression of MMP-2 and MMP-9 induced by TGF-*β*. (a) The positive correlation between TGF-*β* and MMP-2 transcript levels in GSE17538. (b) The positive correlation between TGF-*β* and MMP-9 transcript levels in GSE17538. (c, d) PCR and western blot analysis showed that TGF-*β* induced MMP-2 and MMP-9 mRNA levels in SW480 cells. The treatment of the cells with MnTE-2-PyP markedly inhibited TGF-*β*-induced MMP-2 and MMP-9. ^∗^*P* < 0.05 compared to the control group. (e) Kaplan-Meier curves indicating the overall survival in GSE17538 with low or high levels of MMP-2 (cutoff value = 9.77). (f) Kaplan-Meier curves indicating the overall survival in GSE17538 with low or high levels of MMP-9 (cutoff value = 8.52).

To clarify the mechanism of TGF-*β*-induced migration and invasion, we performed the comprehensive analysis of a public colorectal cancer dataset (GSE17538). Here, we identified various TGF-*β* correlated genes in colorectal cancer. Among them, we discovered matrix metalloproteinases, such as MMP-2 and MMP-9, which are widely involved in tumor metastasis. In line with other studies that TGF-*β* is strongly correlated with MMP-2 and MMP-9, a positive correlation between TGF-*β* and MMP-2 or MMP-9 transcript levels was noted in the publicly available dataset GSE17538 (*R* = 0.554, *P* = 4.6 × 10^−20^ and *R* = 0.477, *P* = 1.4 × 10^−14^, Figures 6(a) and 6(b)). Meanwhile, real-time qRT-PCR and western blot assays indicate that MnTE-2-PyP inhibits TGF-*β*-induced MMP-2 and MMP-9 in colorectal cancer cells (Figures 6(c) and 6(d)). Furthermore, MMP-2 and MMP-9 were closely associated with the poor outcome of cancer patients (Figures 6(e) and 6(f)). In summary, these data demonstrated that MMP-2 and MMP-9 correlate with worse outcomes in colorectal patients and that MnTE-2-PyP inhibits the expression of MMP-2 and MMP-9 induced by TGF-*β*.

## 4. Discussion

Manganese(III) porphyrins (MnPs) have been extensively studied due to their anticancer effects, and they have the ability to protect nontumor tissues from ROS-mediated side effects. Recent studies have shown that the Mn porphyrin analogs MnTnHex-2-PyP(5+) and MnTnBuOE-2-PyP(5+) can modulate the metastatic ability of cancers [19, 20]. A previous study showed that MnTnHex-2-PyP(5+), by suppressing the phosphorylation of several MAP kinases, enhances radiation-induced apoptosis in mouse breast tumor cells [29]. Furthermore, in the inflammatory breast cancer cell line, the involvement of MnTnBuOE-2-PyP(5+) in ERK and NF-*κ*B signaling led to cell death [30]. Recently, our group revealed that MnTE-2-PyP reduces prostate cancer growth and metastasis in combination with radiation [7, 31]. MnTE-2-PyP has also been shown to sensitize tumor tissue to chemotherapy agents [32–34]. In a breast cancer model, MnTE-2-PyP suppresses tumor growth via antiangiogenic effects at the level of tumor vasculature [35]. These studies demonstrate that MnTE-2-PyP is an effective antitumor agent. The present study provides novel evidence that MnTE-2-PyP also inhibits cancer cell progression by reversing the EMT process.

In this study, we showed that MnTE-2-PyP inhibited TGF-*β*-induced expression of EMT markers and morphological changes in colorectal cancer cells. Mechanistically, MnTE-2-PyP attenuated TGF-*β*-induced EMT in colorectal cancer cells by inhibiting the Smad2/3 signaling pathway. However, MnTE-2-PyP did not suppress TGF-*β*-induced slug and snail expression in colorectal cells. Furthermore, MnTE-2-PyP effectively suppressed colorectal cancer cell migration and invasion and the expression of MMP-2 and MMP-9 caused by TGF-*β*.

Interestingly, MnTE-2-PyP did not inhibit TGF-*β*-induced EMT completely in colorectal cancer cells. Extracellular TGF-*β* binds to its two transmembrane receptors (TGF*β*RI and TGF*β*RII), which signal via cytoplasmic Smad proteins and alternative pathways, including MAPK, PI3K/AKT, and GTPase pathways [11–15]. Chatterjee et al. have recently shown in a radiation-induced fibrosis model that MnTE-2-PyP inhibits the fibroblast activation pathway by downregulating the expression of TGF*β*RII, which in turn reduces the activation and/or expression of Smad2, Smad3, and Smad4. In this study, we showed that TGF-*β* induced an increase in phosphorylated Smad2/3 protein levels in SW480 cells. Our results indicated that MnTE-2-PyP attenuated TGF-*β*-induced EMT in colorectal cancer cells by inhibiting the Smad2/3 signaling pathway. One of the major negative regulators of the TGF-*β*-Smad system is Smad7, which has been assumed to serve as a brake of this system [4]. Smad4 and Smad6 are also involved in TGF-*β*-induced EMT [13, 14]. Further studies would aim at determining the effects of MnTE-2-PyP on Smad7, Smad6, and Smad4 in TGF-*β*-induced EMT. In addition, the effects of MnTE-2-PyP on non-Smad pathways, including MAPK, PI3K/AKT, and GTPase pathways, should also be investigated.

Consistent with the important roles of Smads, the loss of epithelial phenotypes and acquisition of mesenchymal features are achieved through a set of transcription factors involving Slug, Snail, Twist, and Zeb1/2 [8–11]. In our study, changes in the expression of Slug and Snail were observed in TGF-*β*-treated SW480 cells. The transcription factors Slug and Snail bind to the E-cadherin promoter and repress E-cadherin expression in epithelial cells, leading to EMT. TGF-*β* induces Slug and Snail expression either through a Smad-dependent mechanism or indirectly through the activation of other transcription factors or relief of repression. We found that MnTE-2-PyP could not suppress TGF-*β*-induced Slug and Snail expression in colorectal cells. Thus, MnTE-2-PyP reverses TGF-*β*-induced EMT mainly through the inhibition of the Smad signaling pathway and not through the Slug and Snail in colorectal cells.

The expression of the gelatinases MMP-2 and MMP-9 is increased in colorectal cancer tissues when compared to concomitant normal tissues [36, 37]. It has been assumed that MMP-mediated E-cadherin disruption is a key step in tubular cell EMT, and loss of E-cadherin is an important cellular event observed during EMT. MMP-9-mediated degradation of E-cadherin promotes head and neck squamous carcinoma cell migration and invasion [38]. In ovarian carcinoma cells, MMP-9 regulates the posttranslational modification of E-cadherin [39]. Furthermore, MMP-2 and MMP-9 are induced by TGF-*β* in SW480 colorectal cancer cells [40, 41]. Thus, we explored the potential role of MMP-2 and MMP-9 in TGF-*β*-mediated EMT in colorectal cancer. A positive correlation between TGF-*β* and MMP-2 or MMP-9 transcript levels was noted in the publicly available dataset GSE17538. Consistent with previous research findings, TGF-*β* treatment of SW480 cells induced MMP-2 and MMP-9 expression. Treatment of the cells with MnTE-2-PyP markedly inhibited TGF-*β*-induced MMP-2 and MMP-9 expression. Furthermore, MMP-2 and MMP-9 were closely associated with the poor outcome of cancer patients. These studies indicate that MnTE-2-PyP may increase E-cadherin expression via inhibiting TGF-*β*-induced MMP-2 and MMP-9.

## 5. Conclusion

In summary, we have shown that MnTE-2-PyP treatment reverses TGF-*β*1-induced EMT in colorectal cancer. Specifically, MnTE-2-PyP inhibits colorectal cancer cell molecular and morphological changes in response to TGF-*β*1 through the Smad2/3 signaling pathway. Furthermore, MnTE-2-PyP effectively suppresses cell migration and invasion caused by TGF-*β*1.

## Data Availability

The datasets used and analyzed during the current study are available from the corresponding author on reasonable request.

## Abbreviations

EMT: Epithelial-mesenchymal transition
TGF-*β*: Transforming growth factor *β*
MnTE-2-PyP: Manganese(III) *meso*-tetrakis-(N-ethylpyridinium-2-yl)
SOD: Superoxide dismutase
MMP-2: Matrix metalloproteinase 2
MMP-9: Matrix metalloproteinase 9
ROS: Reactive oxygen species
ECM: Extracellular matrix.
